# The relationship between the elevation of haemoglobin A1c level, sleep quality and sleep duration in clinically diagnosed pre-diabetic patients in a nationally representative sample

**DOI:** 10.1177/14791641211067421

**Published:** 2022-02-15

**Authors:** Lindsy Kass, Julia C Sanderson, Terun Desai, Rebecca Hurst

**Affiliations:** 1 School of Life and Medical Science, 156605 University of Hertfordshire, Hatfield, UK

**Keywords:** haemoglobin A1c, type 2 diabetes mellitus, sleep quality, sleep duration

## Abstract

**Background/objectives:**

Type 2 diabetes mellitus (T2DM) is one of the most common chronic illnesses in the United Kingdom accounting for approximately 15% of deaths per year. Growing evidence suggests that sleep duration and quality contributes towards this. This study aimed to determine whether there was a significant relationship between the elevation of haemoglobin A1c (HbA1c) level, sleep quality (SQ) and sleep duration (SD) in clinically diagnosed pre-diabetic patients.

**Subjects/methods:**

Following referral from a relevant healthcare professional, participants (*n* = 40) were registered on the National Health Service England, funded Healthier You: National Diabetes Prevention Programme and completed a Pittsburgh Sleep Quality Index questionnaire to evaluate SQ and SD.

**Results:**

A Spearman’s correlation showed an association between HbA1c, SQ and SD measures. A simple linear regression showed a significant large positive association (rs = 0.913, *p* < 0.001) and significant regression (F (1) = 39, *p* < 0.001) with an R2 of 0.842 between HbA1c level and SQ. Additionally, a significant large negative association (rs = 0.757, *p* < 0.001) and significant regression was found (F (1) = 39, *p* < 0.001) with an R2 of 0.570 between HbA1c and SD.

**Conclusions:**

This study suggests a relationship between SQ, SD and the elevation of HbA1c which may contribute towards prevalence of T2DM and may help to increase adherence to diabetes prevention programmes.

## Introduction

Type 2 diabetes mellitus (T2DM) is one of the most common chronic illnesses in the United Kingdom, with 2.9 million recorded diagnosed individuals in 2012, and approximately 15% of deaths per year caused by this illness.^1,2^ Globally, 415 million people are affected by T2DM.^3^ Pre-diabetes is a term used to describe impaired fasting glycaemia (IFG) and impaired glucose tolerance (IGT).^4^ The condition, nevertheless, describes an increased risk of developing T2DM.^5^ While small lifestyle changes in diet and physical activity can reduce the incidence of T2DM by more than 50% for individuals with pre-diabetes,^6–11^ one behaviour that has become highly prevalent over the past few decades is sleep curtailment.

It has been reported that sleep patterns show considerable changes over time, due to social, cultural, behavioural and environmental influences, with approximately one third of the adult population sleeping less than 6 h per night.^12–14^ Long-term sleep loss is a significant risk factor for increased weight, insulin resistance and thus the development of T2DM. Moreover, it has also been found that there is a significant association between sleep deprivation and the later-onset T2DM in women.^15^ Sleep restriction has additionally been correlated with decreases in leptin (appetite suppression hormone), increases in ghrelin (appetite-stimulating hormone) and elevated appetite.^16^ It has also been established that partial sleep deprivation (4 h per night for 6 nights) in 11 men caused impaired glucose tolerance, higher secretion of nocturnal cortisol concentrations, increased sympathetic nervous system activity and decreased leptin secretion.^17^ This further decrease in night-time glucose tolerance is dependent on the occurrence of sleep.^10^ An association between sleep duration and increased risk of the development of T2DM has been reported by researchers^15,18^; however, further research is required to better explain the biological mechanisms underlying this correlation.^15^

Sleep quality, similarly to sleep duration, plays an essential role in maintaining normal physiology.^10^ Sleep fragmentation has been reported as one of the main characteristics of poor sleep quality.^19^ Studies have established that sleep fragmentation without variations in total sleep duration causes abnormal glucose metabolism.^20,21^ Additionally, prospective population-based studies reported an association between poor sleep quality and the incident of T2DM.^18,22,23^ Meta-analyses of these investigations indicated that self-reported decreases in sleep quality predicted the development of T2DM with a relative risk of 1.84 (total participants 24,192).^24^

There is growing evidence that sleep duration (SD) and sleep quality (SQ) are a contributing factor towards the current T2DM epidemic.^15,25,26^ U-shaped patterns have been observed in relationships between SD, SQ, all-cause mortality, obesity and T2DM.^25,27,28^ However, epidemiologic studies on the relationship between SD, SQ and the development of T2DM have shown varying results.^18,25,29^

Rizza et al. (2021)^30^ analysed alterations in HbA1c in both active and former night shift workers with disturbed circadian rhythms to determine premature gluco-metabolic defects. They concluded that apparently healthy young nurses doing rotating night shifts showed mild but significant increases in HbA1c levels compared with diurnal workers, independently of sex, age and BMI. HbA1c levels were also higher in former night shift workers compared with diurnal workers.

Glucose tolerance is critically dependent on the ability of the pancreatic beta cells to release insulin both acutely and in a sustained fashion, and on the ability of insulin to inhibit glucose production by the liver and promote glucose utilization by peripheral tissues, known as insulin sensitivity.^10^ Reduced insulin sensitivity, or insulin resistance, occurs when higher amounts of insulin are required to reduce blood glucose levels following the administrative of the same amount of exogenous glucose.^10^ In healthy individuals, glucose tolerance varies across the day such that plasma glucose response to exogenous glucose is markedly higher in the evening than in the morning, and glucose tolerance is at its minimum in the middle of the night.^30^

Sex differences have been found to affect sleep patterns,^31^ with SQ, SD and latency differing dependent on gender.^32^ Sleep latency is described as the length of time it takes to fall asleep.^33,34^ Longer sleep latency in women has been reported compared with men; additionally, differences have been reported between subjective versus objective measures of SQ between men and women.^32,35^ Reports have stated that normal sleep in women is influenced by hormonal fluctuations and regularly causes sleep fragmentation, with the prevalence of insomnia increasing from 33% in premenopausal women to 61% in postmenopausal women.^36^ In a sex-specific meta-analysis of epidemiological studies, women were demonstrated to be at a 40% increased risk of insomnia.^37^ Additionally,^38^ reports state that decreased SD and reduced SQ correlated with insulin resistance. However, more prospective longitudinal studies are required to further understand the male and female associations between sleep and the development of T2DM.

HbA1c offers an indication of an individual’s average blood glucose levels during the previous 3 months, which is the predicted half-life of red blood cells.^39^ HbA1c was agreed by the American Diabetes Association (ADA) as a diagnostic test for diabetes in 2009^40^ and the World Health Organization (WHO) in 2011.^41^ HbA1c advantages in diagnosis of T2DM include ‘standardization of measurement’,^42^ non-invasive capillary blood-draw that does not involve fasting, negligible day-to-day variability and ‘pre-analytical sample stability’.^42^ Those with HbA1c values 42 mmol/mol–47 mmol/mol are considered by the ADA and WHO to have the highest risk of developing diabetes.^40,41,43^

The aim of this study was to determine whether there is a significant relationship between the elevation of haemoglobin A1c (HbA1c) level, sleep quality (SQ) and sleep duration (SD) in clinically diagnosed pre-diabetic patients in a nationally representative sample in the United Kingdom.

## Methods

The study was conducted between January 2019 and April 2019 with participants on a National Health Service England (NHSE) funded Healthier You: National Diabetes Prevention Programme (NDPP).

### Participants

Forty participants took part in the study. Potential participants were approached by phone call once they were referred and registered on the NHSE NDPP. These programme participants were recruited through healthcare professional referrals (nurses and GPs) and self-referrals.

Participants were eligible if aged 18 years and over; had an HbA1c referral reading of between 42 and 47 mmol/mol within the last 12 months and were registered on the NHSE NDPP. Exclusion criteria included participants with a severe debilitating disease which may have interfered with the study participation, under the age of 18 years, over the age of 65 years and/or pregnant. Participant descriptive characteristics are outlined in Table 1.

**Table 1.** Descriptive characteristics of the study population showing units, mean $\left(\bar{x}\right)$ and standard deviation (SD) where appropriate.

Descriptive characteristic (units)	$\bar{x}$ (±SD)
Age (years)	52.87 (±8.79)
Gender – female/male	26/14
HbA1c (mmol/mol)	44.19 (±1.58)

The University of Hertfordshire Health Science Engineering and Technology Ethics Committee approval was granted (protocol number LMS/UG/NHS/02928 and Health Research Authority REC approval 19/WM/0031). Participants gave written consent to take part in the study, following both written and verbal information beforehand on the study protocol from the researcher. Health screening to determine NDPP suitability was undertaken directly by the intervention provider.

The study was a retrospective observational cohort study, designed to assess the relationship between the elevation of HbA1c level, SQ and SD in clinically diagnosed pre-diabetic participants referred and registered on the NHSE NDPP. Participants received a pre-program information pack in conjunction with a SQ and SD questionnaire at the end of their 1:1 initial appointments for the NHSE NDPP. Participants were given prepaid envelopes to send their anonymized questionnaires back to the researcher once completed after their 1:1 appointment.

### Outcome measures

Sleep quality and SD were assessed using the Pittsburgh Sleep Quality Index (PSQI),^44^ evaluating SQ and SD over a 1-month time interval. The PSQI is a 19-item self-rated questionnaire that generates seven sleep component scores on a 0–3 scale, with three indicating the greatest dysfunction.^44^ The PSQI score was comprised of the sum of the scores for the seven components in a way that a higher score indicated a worse SQ. Poor SQ was defined as PSQI score greater than 5. SD was defined as the number of hours participants spent sleeping.

### Data analysis

The primary outcomes of the study were SQ and SD. All analyses were undertaken using IBM SPSS 22 (SPSS, Chicago, IL, USA). Testing for normality was undertaken: SQ and SD were found not be normally distributed. A Spearman’s correlation was performed between HbA1c, SQ and SD measures. A simple linear regression was calculated to predict HbA1c based on the two significant correlates (SQ and SD). Statistical significance was set at a level of *p* < 0.05 for all tests.

## Results

100% of the participants completed the PSQI questionnaire and other relevant documents, with no dropouts during the study.

### Relationship between HbA1c and sleep quality

**Figure 1.**
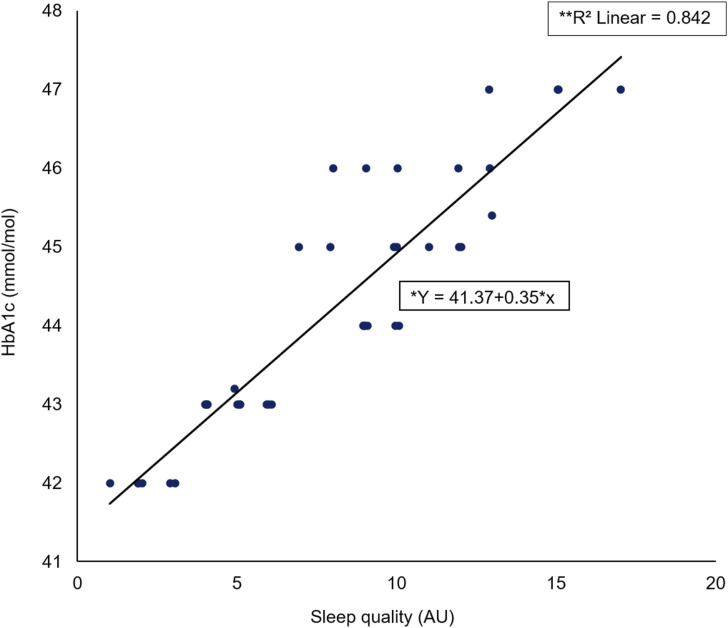
Scatter plot of HbA1c (mmol/mol) against sleep quality (AU) including *regression equation and **slope coefficient.

A Spearman’s rho correlation for the entire cohort reported a significant large positive association (rs = 0.913, *p* < 0.001) between HbA1c elevation level (mmol/mol) and SQ (AU) (Figure 1). Additionally, a significant regression was found (F (1) = 39, *p* < 0.001) with an R^2^ of 0.842.

### Relationship between HbA1c and sleep duration

**Figure 2.**
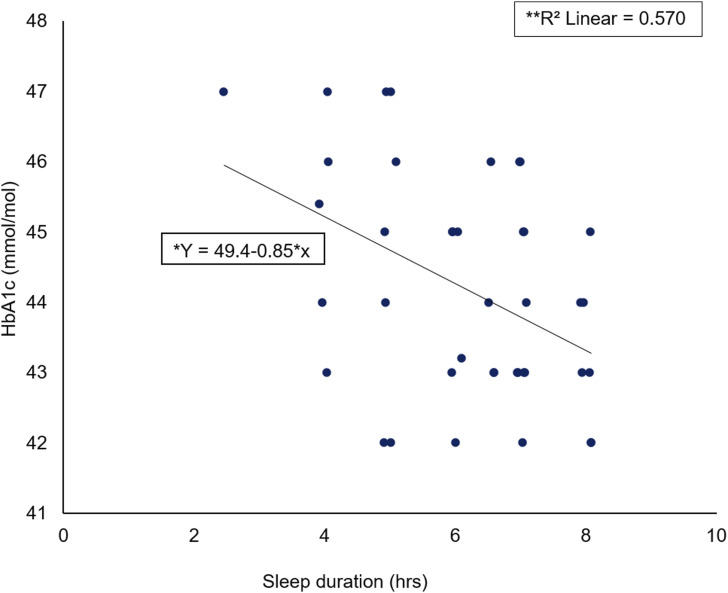
Scatter plot of HbA1c (mmol/mol) against sleep duration (hrs) including *regression equation and **slope coefficient.

A Spearman’s rho correlation reported a significant large negative association (rs = −0.757, *p* < 0.001) between HbA1c elevation level (mmol/mol) and SD (hours) (Figure 2). A significant regression was found (F (1) = 39, *p* < 0.001) with an R^2^ of 0.570.

### Relationship between female HbA1c and sleep quality

**Figure 3.**
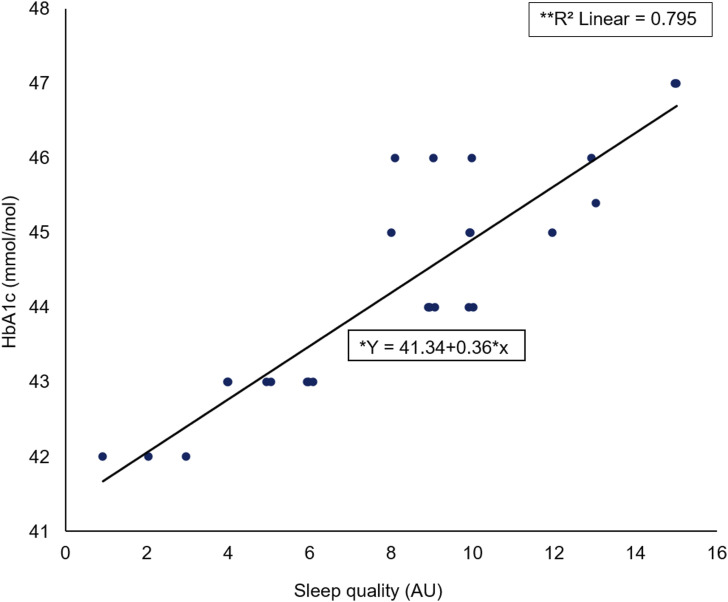
Scatter plot of female HbA1c (mmol/mol) against sleep quality (AU) including *regression equation and **slope coefficient.

A Spearman’s rho correlation reported a significant large positive association (rs = −0.868, *p* < 0.001) between female HbA1c elevation level (mmol/mol) and SQ (AU) (Figure 3). A significant regression was found (F (1) = 25, *p* < 0.001) with an R^2^ of 0.795.

### Relationship between female HbA1c and sleep duration

**Figure 4.**
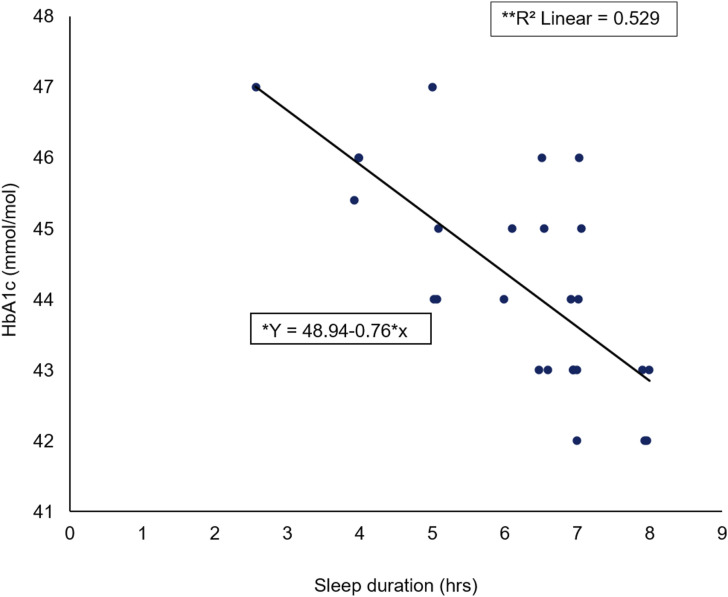
Scatter plot of female HbA1c (mmol/mol) against sleep duration (hrs) including *regression equation and **slope coefficient.

A Spearman’s rho correlation reported a significant large negative association (rs = −0.723, *p* < 0.001) between female HbA1c elevation level (mmol/mol) and SD (hours) (Figure 4). A significant regression was found (F (1) = 25, *p* < 0.001) with an R^2^ of 0.529.

### Relationship between male HbA1c and sleep quality

**Figure 5.**
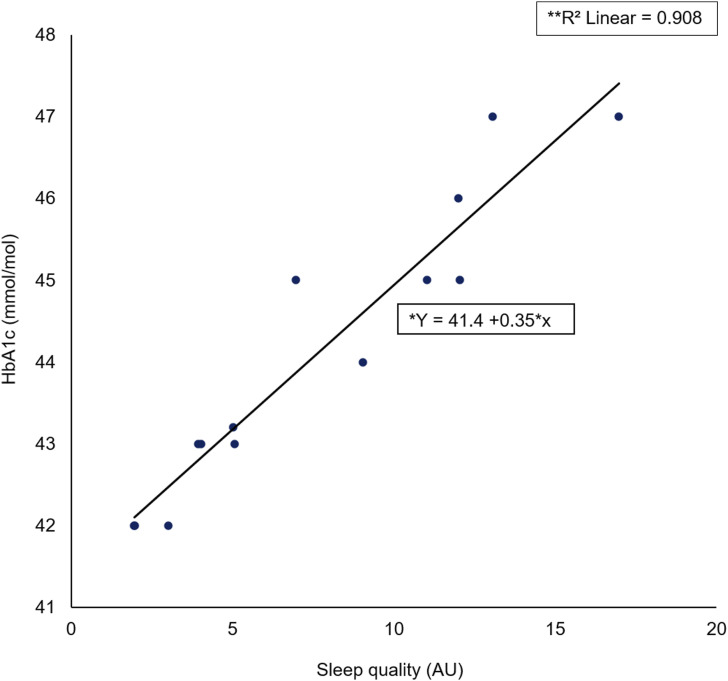
Scatter plot of male HbA1c (mmol/mol) against sleep quality (AU) including *regression equation and **slope coefficient.

A Spearman’s rho correlation reported a significant large positive association (rs = −0.972, *p* < 0.001) between male HbA1c elevation level (mmol/mol) and SQ (AU) (Figure 5). A significant regression was found (F (1) = 13, *p* < 0.001) with an R^2^ of 0.908.

### Relationship between male HbA1c and sleep duration

**Figure 6.**
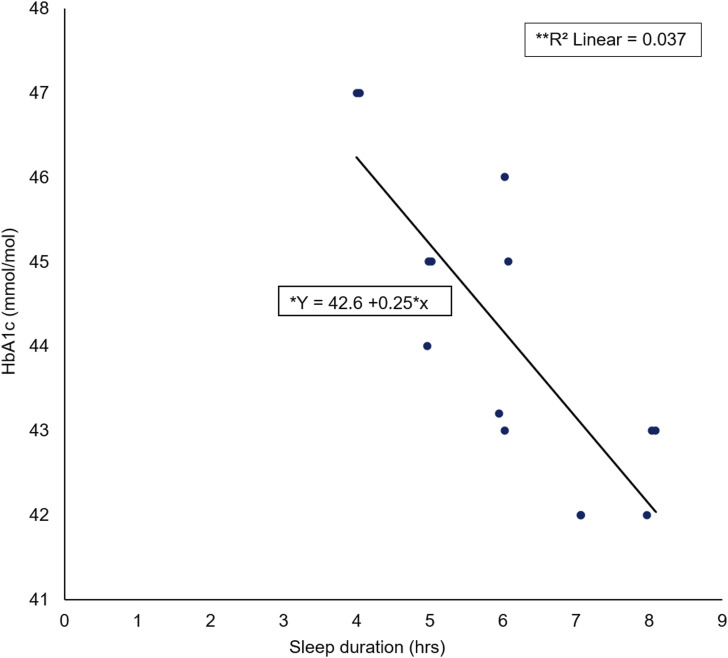
Scatter plot of male HbA1c (mmol/mol) against sleep duration (hrs) including *regression equation and **slope coefficient.

A Spearman’s rho correlation reported no significant association (rs = 0.218, *p* = 0.454) between male HbA1c elevation level (mmol/mol) and sleep duration (hours) (Figure 6). No significant regression was found (F (1) = 13, *p* = 0454) with an R^2^ of 0.037.

## Discussion

The main findings emerging from this study were that those participants with a higher HbA1c elevation level had a higher global PSQI score/decreased SQ. Secondly, it was shown that an increase in SD resulted in a decreased HbA1c elevation level. The secondary findings emerging from the study found that females with a higher HbA1c elevation level had a higher global PSQI score/decreased SQ; additionally, increases in SD resulted in a decreased HbA1c elevation level for female participants. Furthermore, males with a higher HbA1c elevation level had a higher global PSQI score/decreased SQ; however, no association between male HbA1c elevation level and SD was reported.

There are several contributing pathways to abnormal glucose metabolism as a consequence of sleep restriction.^19^ Brain glucose utilization was observed to be considerably reduced during total sleep deprivation^45^; furthermore, following sleep restriction, prolonged nocturnal growth hormone secretion can cause morning insulin resistance.^46^ Increases in inflammatory markers because of sleep restriction have been well documented.^47,48^ These include ‘cytokine interleukin-1β’,^49^ ‘Interleukin 6’,^50^ Interleukin 17A’,^51^ ‘tumour necrosis factor alpha’,^52^ ‘high-sensitivity C-reactive protein’^53^ and increased ‘leukocytes and monocytes’.^54,55^ It has also been reported that these inflammatory markers are associated with insulin resistance.

Previous studies have found that self-reported short SD and decreased SQ is associated with the development of T2DM.^22,26,56,57^ In particular, Lou et al. (2012) have shown in a large population sample of Chinese adults that decreased SQ and decreased SD were found to be significantly associated with an increased risk of the development of T2DM. Although working with a different specialist population, the findings of the present study agree with these findings, suggesting that SD and SQ could be a contributing factor towards the development of T2DM.

When focusing specifically on gender, SQ, SD and the development of T2DM, there have been fewer studies that have been undertaken on the associations between these variables.^31,32,34^ Female results in the present study found a correlation between increased PSQI scores and decreased SD, indicating that gender might be a contributing factor to the development of T2DM; furthermore, the prevalence of pre-diabetes differs between sexes.^58^ Report shows that men more frequently develop IFG,^58^ whereas women more frequently develop IGT have also been found. In a meta-analysis, both decreased SD (<5 h) and problems initiating or maintaining sleep were correlated with higher diabetes risk.^24^

Insulin resistance plays a major role in the development of T2DM.^59^ Thus far, there have been 14 studies on the influences of sleep restriction on the metabolism of glucose.^17,46,60–71^ In a breakthrough study, researchers found that the effects of six nights of 4 h restricted sleep on glucose metabolism, measured by ‘intravenous glucose tolerance testing’,^17^ caused a 24% decrease in insulin sensitivity and a 30% decrease in acute insulin response to glucose, indicating an insufficient β-cell response to increased insulin resistance.^17,72^ There have been reports that the differences in glucose metabolism were supplemented by a difference in ‘cardiac sympatho-vagal activity’.^72^ Further studies have similarly evaluated the influences of restricted sleep on glucose metabolism using various methods including ‘morning fasting levels’,^62^ repeated sampling throughout the daytime^61^ and additional ‘dynamic testing’^60^ Although there were differences in methodology, the majority of the studies reported adverse effects of restricted sleep on glucose metabolism.^19^ This could explain why the participants with decreased SQ and SD in the present study had an increased HbA1c elevation level.^73^ Long-term, insufficient sleep may be a factor promoting the development of T2DM.

The prevalence of T2DM in England grew from 2.3 million in 2009/2010 to 3.0 million in 2015/2016.^74^ Furthermore, it is predicted that the prevalence will increase to approximately 5.6 million individuals with T2DM by 2035/2036.^75^ The total cost of direct patient care for T2DM in the NHS during 2010–2011 was estimated at £9.8 billion, whilst the indirect costs were estimated at £13.9 billion.^76^ It has been estimated that the cost burden of diabetes in 2010/2011 was 10% of the total NHS resource expenditure.^77^ Moreover, it has been reported that if no changes are made, this will increase to an estimated 17% of NHS expenditure by 2035/2036.^77^

## Study limitations and ideas for future research

The PSQI questionnaire is a subjective measure of sleep and self-reporting can reflect inaccurate information if the participant had difficulty understanding the questions.^78^ Future research should look at using more accurate measures of SQ and SD to ensure accurate representations of both sleep restriction and sleep fragmentation. Additionally, no other anthropometric risk factors were measured, due to The Healthier You: NHS Diabetes Prevention Programme currently being a pilot study, and no anthropometric risk measurements recorded by the intervention provider were able to be used in the current study. It would therefore be advantageous to measure SQ and SD adjunct to anthropometric measurements such as body mass index and waist measurements.

## Conclusion

In conclusion, a significant relationship was found between the elevation of HbA1c level, sleep quality and SD in clinically diagnosed pre-diabetic patients in a nationally representative sample. Future research should look at using more accurate measures of sleep quality and SD to ensure accurate representations of both sleep restriction and sleep fragmentation; additionally, it would be advantageous to measure sleep quality and SD adjunct to anthropometric T2DM risk factor measurements, such as body mass index and waist circumference.

Availability of data and material can be accessed at the University of Hertfordshire’s repository at https://doi.org/10.18745/ds.24981.
